# Neurology

**Published:** 1894-09

**Authors:** William C. Krauss


### NEUROLOGY.

By WILLIAM C. KRAUSS, M. D.

### JACKSONIAN EPILEPSY DUE TO AUTO-INTOXICATION OF GASTRIC ORIGIN.

IN THE *Rivista Sperimentale di Freniatria edi Medicina Legale*, Vol. XIX., fas. iv., Doctor Cristiani describes a very interesting case of Jacksonian epilepsy, due to gastric intoxication. The patient was a man 52 years old, strong, healthy, with good family history, no history of vices or excesses ; no disturbances of any of the sensory organs, no arterio-sclerosis, no malformation of head or scar on scalp ; urine normal in quantity and quality. For some years he has suffered from stomach and intestinal disorders, such as loss of appetite, nausea, ructus, pyrosis, tongue coated, mouth dry and bitter, bowels constipated. Occasionally, besides these symptoms he would notice paranesthesia of the limbs, flushings, then coldness of the extremities, and such mental symptoms as depression, melancholia, hypochondriasis, irritability, cephalalgia and vertigo.

On the morning of September 8, 1892, he had the first attack. The aura was distinctly felt, consisting of a feeling of heat in the head, anxiety and precordial oppression. He then felt creeping sensations in the arm and leg of the right side, followed by clonic spasms first of the right arm then of the right leg, and at the acme of the attack the right side of the face would become involved. These paroxysms would last from five minutes to fifteen minutes, with no loss of consciousness, would not fall to the ground, but, in a vaccillating manner, stagger to the right. On the first day he had three attacks, then about one attack every other day for two weeks. The pupils were dilated, patellar reflex exaggerated on the right side, and urine contained an excess of urea and phosphates. The treatment directed to the gastric catarrh had the effect of controlling the attacks, and in over a year they have not reappeared.

---

### THE SPECIFIC GRAVITY OF THE URINE OF THE INSANE.

AFTER reviewing the labors of several alienists on this point and the variance in their results, Dr. Umberto Stefani, of Padova, made a careful investigation, and studied for one or more months sixty cases of mental diseases, with the following results: In all cases of acute insanity, independent of special forms, the specific gravity ranged from 1030 to 1040, and sometimes higher. If the form is of short duration, the specific gravity will fall as the psychic symptoms diminish, until it reaches the normal, or even lower. When the remission of the psychic symptoms is followed by an exacerbation, the specific gravity of the urine will again increase.

If the course of the disease is long and tends to become chronic, the density of the urine ordinarily will, after a time, commence to diminish until it reaches normal or falls below. In cases of imbecility, paranoia, senile dementia and paresis without spells of excitement, the specific gravity of the urine is not increased. But if continued, frenzy and excitement develop, the specific gravity will increase to 1030–1040.

The writer throws out the suggestion that, in the examination of the urine, we have an index, probably, of the prognosis and course of the disease. A table of charts, showing the exacerbations and remissions, adds to the interest of the paper.—*Rivista Sperimentale di Freniatria edi Medicina Legale*, Vol. Xp., fas. i., 1894.

---

### A STUDY OF THE REFLEXES IN THIRTY-FOUR EPILEPTICS.

DR. ARTHUR DOUAGGIO, of Reggio, publishes in the *Rivista Sperimentale di Freniatria edi Medicina Legale*, fas. i., 1894, the results of his examination of the reflexes, muscular force and measurements of the extremities in 34 cases of epilepsy. In the interval he found the patellar reflexes exaggerated in 15, medium in 8, diminished in 7 and absent in 2 cases. There existed in the 15 cases a difference of exaggeration, being stronger on the right side in 10, stronger on the left in 5 cases. The tendo Achilles reflex in 27 cases was exaggerated in 9, medium in 13, diminished in 3 and absent in 2 cases. The bicipital in 31 cases was exaggerated in 8, medium in 12, diminished in 9 and absent in 2 cases.

The olecranon reflex was exaggerated in 6, medium in 9, diminished in 9 and absent in 7 of 31 cases examined. The abdominal reflex in 32 cases was exaggerated in 3, medium in 3, diminished in 11 and absent in 15 cases. The cremasteric reflex in 25 cases examined was exaggerated in 2, medium in 8, diminished in 7 and absent in 8 cases. Pupillary reflexes, examined in reference to pain, reacted promptly in 10 cases, medium in 9, slowly in 4 and not at all in 7 cases of 30 examined. In regard to the light reaction, prompt in 5, medium in 13, slowly in 12 cases. In reference to the inequality of the pupils, the author found in 4 cases only a slight inequality.

